# Supplementary material for: Scaling laws for function diversity and specialization across socioeconomic and biological complex systems
Source: Proc Natl Acad Sci U S A. 2026 Feb 12;123(7):e2509729123. doi: 10.1073/pnas.2509729123 (PMC12912993; doi:10.1073/pnas.2509729123)
Supplement: Supplementary file 1 — Appendix 01 (PDF) [file pnas.2509729123.sapp.pdf]

PNAS

Supporting Information for

Scaling Laws for Function Diversity and Specialization Across Socioeconomic and Biological Complex Systems

Vicky Chuqiao Yang, James Holehouse, Hyejin Youn, José Ignacio Arroyo, Sidney Redner, Geoffrey B. West and Christopher P. Kempes

Corresponding authors: V.C. Yang (vcyang@mit.edu); J. Holehouse (jamesholehouse1@gmail.com)

This PDF file includes:

Supporting text
Figs. S1 to S11
Tables S1 to S2
SI References

Supporting Information Text

Contents

1 Data **2**

2 Data Analysis Methods **3**

3 Additional Empirical Results **3**

4 Supporting Material for Calibration Procedure **6**

A Data Preparation 6

B The Calibration Algorithm 6

C Pseudo-code for Monte Carlo Simulation of Organization Growth 6

D Estimating the Value of p_0 from Diversity Curves 7

E Reducing Rank-Frequency Distribution Size for Large Organizations 7

5 Additional Modeling and Calibration Results **10**

6 Robustness Analyses **14**

A Diversity Scaling of Urban Areas Across Multiple Years 14

B Stability of Estimated Parameters over Multiple Years of Data 14

C Verify Model's Dynamic Predictions Using Longitudinal Data 14

D Robustness Analysis of the Calibration Procedure 14

1. Data

Prokaryotic Cells. A majority of the proteome data was obtained from pax-db.org Version 5.0 https://pax-db.org/ (1) in addition to other datasets obtained from the literature. The compiled database is available in our replication folder.

The Proteome dataset includes almost the whole range of cell sizes found in bacteria, from the smallest prokaryotes, which commonly are obligate symbionts, to one of the largest free-living species, obtained from the Protein Abundance Database (2). The range in genome size varied from 0.19 Mbp (in *Sulcia muelleri*) to 11.74 Mbp (in *Sorangium cellulosum*). From this data set we extracted the relationship between the total number of protein types and the number of protein molecules in the cell. Dividing the total protein volume by the average volume of an individual protein, we calculated the average number of protein molecules per cell for a given species. To calculate the number of protein molecules, we used well-supported scaling relationships between DNA, proteins, and cell volume. Cell volumes are estimated from genome length in Mbp based on the reference genomes at NCBI at https://www.ncbi.nlm.nih.gov/genome/, using the relationship between cell and genome size (3, Figure 1); $V = V_0 G^a$, where V is the cell volume, V_0 is an inferred constant and G is the DNA content. From cell volume, we estimated total protein volume based on well-supported scaling relationships between protein volume and cell volume (4, 5); $P = P_0 V^b = P_0 (V_0 G^a)^b$, where P is the protein content and P_0 is an inferred constant. Then to calculate the number of protein molecules in the cell, N_{cell}, we divide the total protein volume P, by the calculated average protein volume, P_{prok}, based on the average amino acid volume and the average protein length in prokaryotes); $N_{\mathrm{cell}} = P/P_{\mathrm{prok}}$. In our analysis, we only included data from samples with a coverage (ratio of expressed to encoded proteins) above 0.5. The methods for proteomic scaling data are described in detail in (6).

US Federal Agencies. The data on US Federal agencies are compiled from the FedScope Employment Cube provided by the US Office of Personnel Management (OPM). The data can be accessed at https://www.fedscope.opm.gov/employment_access.asp. The data used in our analysis are from September 2018. The dataset contains 125 cabinet-level departments and independent agencies. The smallest agencies in this dataset contain two employees, which are the Commission for the Preservation of America's Heritage Abroad and the Northern Border Regional Commission. The largest agencies contain hundreds of thousands of employees—the largest two being the Department of Veterans Affairs, with 391,187 employees, and the Department of the Army, with 249,074 employees. The FedScope data is compiled through human resource software used by the US federal government. Note that not all federal agencies are reported in this data set; however, all agencies available through FedScope are used in our analysis.

The size of a federal agency is defined as the number of total employees. Functions of the employees are defined according to the OPM's Handbook of Occupational Groups and Families, available at https://www.opm.gov/policy-data-oversight/classification-qualifications/classifying-general-schedule-positions/occupationalhandbook.pdf. The occupation categories used in our analysis are on the 4-digit level, the finest level in the dataset.

Universities. The data on US universities are obtained from the Integrated Postsecondary Education Data System (IPEDS). The data was accessed from https://nces.ed.gov/ipeds/use-the-data. The data used in our analysis are from the year 2016. Our analysis for bachelor-level and above institutions includes universities classified as "Baccalaureate," "Master," or "Doctoral"

universities in the Carnegie 15 classification system. The associate-level universities are those classified under "Associate" in the Carnegie 15 classification system.

The size of a university is measured by its number of full-time faculty. The function diversity of the faculty is approximated by the number of distinct academic programs under which at least one degree was granted in the year the data was collected. Academic programs are classified by the Classification of Instructional Programs (CIP) code, developed by the U.S. Department of Education's National Center for Education Statistics (NCES). In our analysis, we filter for universities with a minimum of 10 academic programs.

Companies. The data on Norwegian companies were purchased from Statistics Norway in May 2022. The data are from the year 2019. For confidentiality, the company-level data was aggregated by Statistics Norway according to the following procedure. The companies are first sorted by the number of employees from large to small. For every five companies, both the number of employees and the number of distinct functions are aggregated by taking the mean of the log-transformed variables, i.e., $\mathrm{E}[\log y_i]$ where y_i is a variable for company i in that size bin. For confidentiality reasons, this data only includes companies with five or more employees. The functions are defined by the 4-digit International Standard Classification of Occupations (ISCO-08), which is detailed at https://www.ssb.no/en/klass/klassifikasjoner/7/versjon/33. Note that this dataset only includes resident employees in Norway, and it does not include subsidiary companies in countries other than Norway.

Urban areas. The data on occupations in US urban areas are obtained from the US Bureau of Labor Statistics (BLS). The data were accessed from https://www.bls.gov/oes/tables.htm. In our analysis, we use data from the year 2017. The occupation classification is according to the 2010 Standard Occupational Classification (SOC) system (https://www.bls.gov/soc/). The boundaries of urban areas in this dataset use the Metropolitan Statistical Area established by the US Office of Management and Budget (OMB).

2. Data Analysis Methods

Code and data used for our analysis are available online at: https://github.com/jamesholehouse/Calibrations-for-Function-Diversity.

Fitting power law exponents. Consistent with previous literature on scaling in human social systems (7), we obtain the scaling exponent of the data by performing ordinary least square regressions on the log-transformed variables. In other words, estimating parameters β and c in $\log Y_i = \beta \log X_i + c$, where X_i and Y_i are the data of dependent and independent variables, and β is the scaling exponent. The estimation uses ordinary least square regression, and the confidence interval is based on Student's t-distribution. Zeros and missing values are removed from the datasets before making the logarithmic transformation.

Estimation methods for cells. The methods for proteomic scaling data are described in detail in (6). In summary, we estimated cell volume from genome length in Mbp based on the reference genomes at NCBI at https://www.ncbi.nlm.nih.gov/genome/, based on the relationship between cell and genome size (3). From cell volume, we estimated total protein volume based on well-supported power-law relationships in the literature (4, 5). Then, considering an average protein volume (based on the average amino acid volume and the average protein length in prokaryotes), by dividing the total protein volume by a typical individual volume, we estimated the number of protein molecules in the cell. To get the scaled individual protein abundances N_s, each observed protein abundance (N_i) for a sample was multiplied by the ratio of predicted (N_p) to observed total abundance (N_t), i.e., $N_s = N_i(N_p/N_t)$. In this calculation, the number of peptides in a sample could be lower or higher than the amount per cell, but there is a correction analogous to rarefaction to achieve to predicted total. This assumes that in the sampling process, not each peptide of each protein is characterized, and then there is a sampling effort curve. We only included data of samples with a coverage (ratio of expressed to encoded proteins) above 0.5 (see (6)).

3. Additional Empirical Results

See Fig. S1 for additional empirical results for the scaling relationship between size and function diversity for Norwegian companies, US universities at bachelor level and above, and US universities at associate level.

See Table S1 for additional statistics on the scaling exponent for the number of distinct functions, including t-tests assessing whether the exponent differs significantly from zero.

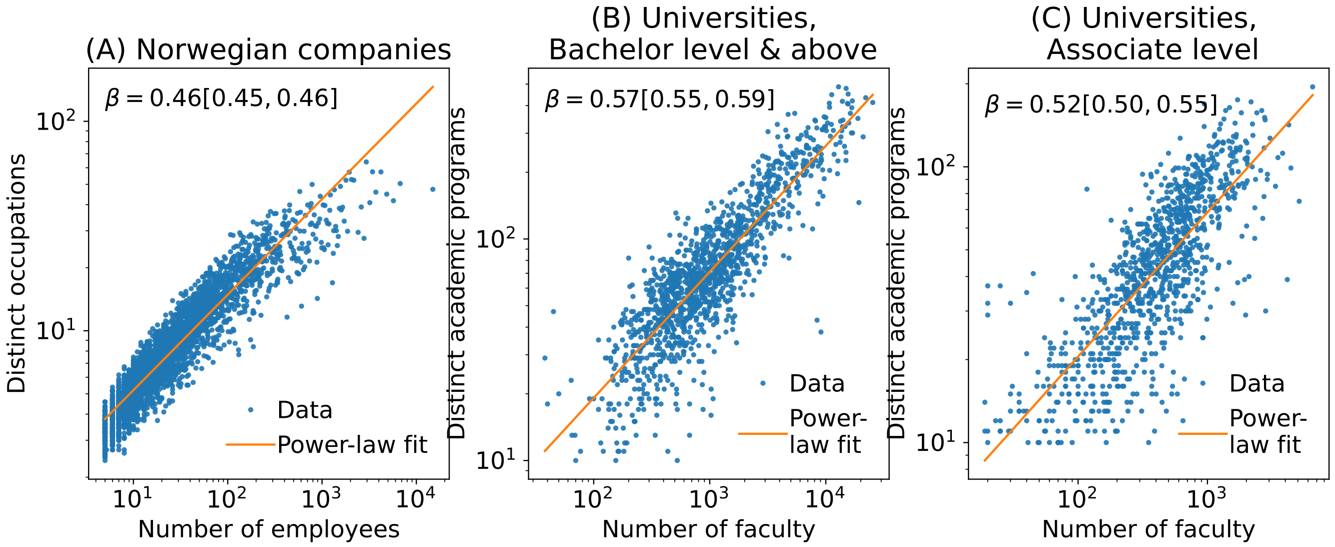

Fig. S1. The scaling relationship between size and function diversity for Norwegian companies, US universities at bachelor level and above, and US universities at associate level.

Table S1. Summary of additional scaling statistics for function diversity in various biological and social complex adaptive systems

	Standard error	t	p-value
Bacteria	0.021	16.8	0.000
Norwegian companies	0.003	152	0.000
US federal agencies	0.017	29.0	0.000
US universities, associate level	0.013	41.0	0.000
US universities, bachelor level & above	0.009	63.3	0.000

4. Supporting Material for Calibration Procedure

In this Section, we describe the stochastic algorithms, mathematical results, data processing and calibration procedure necessary to recapitulate Figures 3 and 4 in the main text. Note that the Sections C, D and E below detail more detailed aspects of the algorithm that are referenced to in this Section.

A. Data Preparation. We began by collecting raw data for all three organizational types, as detailed in Section 1. The data were then assessed based on two criteria: (1) the level of detail and (2) the system size. These factors dictated the subsequent data processing steps.

For (1), the city data appeared to be rounded to the nearest multiple of 10, as individual occupation counts were reported in increments of 10. To account for this, we coarse-grained the data during calibration, treating each addition to the system as representing 10 workers. No systematic rounding was evident in the datasets for federal agency workers or prokaryotic proteomes.

Regarding (2), some systems in all three datasets were large enough to significantly increase simulation time, making direct calibration infeasible. To address this, we developed an algorithm based on the mathematical solution of our model, from (8), to systematically reduce the size of systems exceeding 5×10^5 agents. This threshold was chosen to ensure the optimization algorithm (introduced below) could converge within a runtime of two days. Calibrations were performed on the University of New Mexico's CARC supercomputer, with each system run on a node equipped with 20 GB of RAM. The size reduction algorithm is detailed in Section E.

B. The Calibration Algorithm. We employed a likelihood-free inference method to obtain point estimates for the model parameters $\theta^\star$ and $\gamma^\star$, which best fit the processed data of individual organizations. We consider the parameter p_0 to be consistent across systems of the same type (e.g., all federal agencies have the same p_0 value, all cities have another p_0 value), as it represents the characteristic space of possible functions for that system class. Section D outlines the procedure for estimating p_0 from diversity curves and specifies the p_0 values used in simulation of each system class.

The calibration procedure is outlined below:

1. **Data Import and Preprocessing:** Import the data for a single organization and construct its rank-frequency distribution. Determine the total number of workers from the data—this defines the organization size to be regenerated using the Monte Carlo simulation. Additionally, import the initial conditions from which the simulations will be initiated (specified in Section C).

2. **Parameter Space Definition and Simulation:** Define the bounds of the parameter space to be explored: $\theta \in [\theta_{\min}, \theta_{\max}]$ and $\gamma \in [\gamma_{\min}, \gamma_{\max}]$. For each organization, if the size exceeds 5×10^5 agents then reduce the size of the organization, as specified in Section E. Subsequently, simulate an organization of the required size using these parameters. The parameter space is sampled using the `adaptive_de_rand_1_bin_radiuslimited` algorithm, a type of adaptive differential evolution algorithm (9), implemented via the Julia package `BlackBoxOptim` (10).

3. **Cost Function Evaluation:** Compute the cost function for each parameter combination (θ, γ). The cost is defined as the Euclidean distance in log-space between the normalized rank-frequency distributions of the Monte Carlo simulations (see Section C) and the processed empirical data. The adaptive differential evolution algorithm minimizes this distance by iteratively refining the sampled parameter space to identify the optimal $\theta^\star$ and $\gamma^\star$.

4. **Optimization and Parameter Selection:** Execute the adaptive differential evolution algorithm for a predefined number of iterations, $\mathcal{S}$. Upon completion, record the parameter values $\theta^\star$ and $\gamma^\star$ that minimize the cost function.

Parameter Space Boundaries:
To ensure the inference procedure does not select parameter values near the boundaries, we carefully chose the bounds for the parameter space. For prokaryotic cells, the bounds were set to $\theta_{\min} = 0.0$, $\theta_{\max} = 1.2$, $\gamma_{\min} = 0.0$, and $\gamma_{\max} = 1.2$. For federal agencies, we selected $\theta_{\min} = 0.8$, $\theta_{\max} = 1.5$, $\gamma_{\min} = 0.5$, and $\gamma_{\max} = 1.2$. For cities, the bounds were $\theta_{\min} = -0.5$, $\theta_{\max} = 1.2$, $\gamma_{\min} = 0.0$, and $\gamma_{\max} = 1.0$.

C. Pseudo-code for Monte Carlo Simulation of Organization Growth. We describe here the Monte Carlo algorithm used to calibrate the data to the mechanistic model. This algorithm is a type of stochastic simulation algorithm, commonly known as a Gillespie algorithm, widely applied in stochastic chemical kinetics (11). The procedure is as follows:

1. Initialize the organization with an initial set of functions. Choose a fixed value of p_0 and specify values for the parameters θ and γ. Let $\vec{S}$ be a vector of length D, where D denotes the total number of distinct functions (diversity) within the organization. Each element $[\vec{S}]_i$ represents the number of workers in function i. The total number of workers in the organization is given by $W = \sum_i [\vec{S}]_i$.

2. Sample values 0 and 1 with weighted probabilities $(1 - p(N, \theta))$ and $p(N, \theta)$, where $p(N, \theta)$ represents the probability of introducing a new role based on system size N and the diversification parameter θ. If value 0 is selected, add a new worker to an existing function (step 3); otherwise, assign the worker to a new role (step 4).

3. Add a new worker to an existing function. Sample from the vector $\vec{R} = (1, 2, \ldots, D)$, where each function R_i is selected with a probability proportional to $([\vec{S}]_i)^\gamma$, reflecting a preferential attachment mechanism favoring larger functions. Update $\vec{S}$ according to the rule $[\vec{S}]_{R_i} \leftarrow [\vec{S}]_{R_i} + 1$. Proceed to step 5.
4. Add a new worker to a new role by appending 1 to $\vec{S}$. Proceed to step 5.
5. If $W = M$, where M is the desired organization size, stop and return $\vec{S}$. Otherwise, repeat from step 2.

Initial Conditions: Initial conditions were selected from smaller examples of each system type to capture the expected diversity at the onset of the organizational growth process. The following initial conditions were used in the calibration procedures:

- **Federal Agencies:** The smallest federal agency exceeding 1,000 employees, the National Credit Union Administration $(1,111$ employees), was used as the initial condition. Approximately 40 federal agencies exceeded this size threshold.
- **Cities:** A mid-sized city, Waco, TX, with approximately 100,000 workers, served as the initial condition. This was evolved to match the sizes of the largest 20 cities in the dataset, each with populations exceeding 10^6 workers.
- **Prokaryotic Cells:** The smallest prokaryotic cell in terms of protein abundance, *Nanoarchaeum equitans* (∼25,000 proteins), was used as the initial condition to evolve the remaining 46 protein abundance rank-frequency distributions in the dataset.

D. Estimating the Value of p_0 from Diversity Curves. To estimate the value of p_0 across organizations of the same type, it is essential to first understand the expected growth of diversity within these systems. If an organization begins with an initial diversity $D(N_0)$, the diversity at a larger size N is given by:

$$D(N) = D(N_0) + \int_{N_0}^{N} p(\theta, N')\, \mathrm{d}N'. \qquad [1]$$

This expression indicates that the diversity at system size N equals the initial diversity plus the cumulative probability of new function additions for all system sizes $N' \in [N_0, N]$. From the mathematical solution of the model, it can be shown that the probability of adding a new function scales with system size as $p \sim N^{1/(2-\theta)}$ (8). Substituting this relation into Eq. 1 and integrating yields:

$$D(N) = \left[\frac{p_0(2-\theta)}{\alpha(1-\theta)}\right] N^{\frac{1-\theta}{2-\theta}} + D(N_0) - \left[\frac{p_0(2-\theta)}{\alpha(1-\theta)}\right] N_0^{\frac{1-\theta}{2-\theta}}, \qquad [2]$$

where α is the proportionality constant in the relation $\sum_k k^\theta n_k \sim \alpha N^{1/(2-\theta)}$ where n_k is the number of functions of size k.

To estimate p_0, the functional form of $D(N)$ can be compared to empirical data, which is modeled as a power-law (or logarithmic) fit, $D(N) = aN^b + c$ (or $D(N) = a\ln(N) + c$), as shown in Figure 1 in the main text. The scaling exponent θ is given by $\theta = 2 + \frac{1}{b-1}$, and p_0/α is given by ab.

Next, the initial condition distribution of functions $n_k(N_0)$, as specified in the previous section, is used to determine the value of α through:

$$\alpha \approx \frac{\sum_k k^\theta n_k(N_0)}{N_0^{1/(2-\theta)}}. \qquad [3]$$

Using this value of α, the parameter p_0 can be determined. The estimated values are:

- $p_0 = 35.9$ for prokaryotic cells,
- $p_0 = 2.0$ for federal agencies,
- $p_0 = 111.9$ for cities.

E. Reducing Rank-Frequency Distribution Size for Large Organizations. Simulating rank-frequency distributions for large systems ($N > 10^6$) is computationally intensive and often infeasible with standard Gillespie simulations (11). To address this challenge within our computational constraints, there are two potential approaches: (i) employing computational approximations, such as *tau-leaping* methods (12), which approximate multiple simulation time steps simultaneously, and (ii) systematically reducing the rank-frequency distribution size to facilitate efficient simulation and calibration.

Leveraging specific mathematical properties inherent in the model (8), we adopt the second approach, enabling systematic reduction of large organizations, alleviating computational demands.

The mathematical properties that allow for this reduction are as follows:

1. Diversity scales with system size as $D(N) \sim N^{(1-\theta)/(2-\theta)}$ (8).
2. The *normalized rank-frequency distributions* become independent of system size when sub-linear preferential attachment governs the addition of new functions (8).

A normalized rank-frequency distribution ensures comparability across systems of different sizes by scaling the rank r relative to diversity $D(N)$, such that $\tilde{r} = r/D(N)$. If $k(\tilde{r}, N)$ represents the normalized frequency (i.e., the frequency divided by the system size) of a function with relative rank $\tilde{r}$ in a system of size N, the normalization condition is:

$$\int_0^1 k(\tilde{r}, N)\,\mathrm{d}\tilde{r} = 1.$$

We now utilize these properties in two ways. First, consider the diversity at the original system size N and at the initial condition N_0. Defining $b = (1-\theta)/(2-\theta)$, we can express the diversity as:

$$D(N) = aN^b + c \quad \text{and} \quad D(N_0) = aN_0^b + c,$$

where a and c are constants to be determined. Solving these equations allows us to compute the diversity at a reduced system size $N_0 < N_{\mathrm{red}} < N$:

$$D(N_{\mathrm{red}}) = D(N_0) + \frac{\left[N_{\mathrm{red}}^b - N_0^b\right]\left[D(N) - D(N_0)\right]}{N^b - N_0^b}. \qquad [4]$$

Here, $D(N_{\mathrm{red}})$ is the diversity of the reduced system of size N_{red}, given a value of θ. We can now use the second property, since we can calculate the number of distinct elements for a system of size N_{red}, we can start assigning the normalized frequencies of the reduced system such that $k(\tilde{r}, N_{\mathrm{red}}) \sim k(\tilde{r}, N)$. Here, the "$\sim$" symbol implies that $k(\tilde{r}, N_{\mathrm{red}})$ is assigned the value of normalized frequency for the closest match between $\tilde{r}$ in each system—necessary since the possible values of $\tilde{r}$ in a system of size $N_{\mathrm{red}} \neq N$ and N will not exactly match. Note that this method is more accurate the closer N and N_{red} are to each other. An example of the method is provided in Figure S2 for the case of $\gamma = \theta = 0$, a good test case since the system diversifies quickly as N increases (since θ is small).

There are then two practical points. First, the reason we can assume sub-linear preferential attachment comes from conducting inference on other systems of the same type that are not too large to calibrate without reducing the system size; in every case, sub-linear preferential attachment was observed. Second, in the calibration procedure detailed in Section 4, we rescale the rank-frequency distributions from the data using an estimated value of θ in step 2 of the algorithm and simulate a system of size N_{red} with the same θ. The Euclidean distance between the two normalized rank-frequency distributions is then calculated in log-space, consistent with standard calibration methods that do not require size reduction.

This size reduction method was primarily applied to data from prokaryotic cells, though it was also used for certain federal agencies. In all cases, the reduced system size was set to $N_{\mathrm{red}} = 5 \times 10^5$.

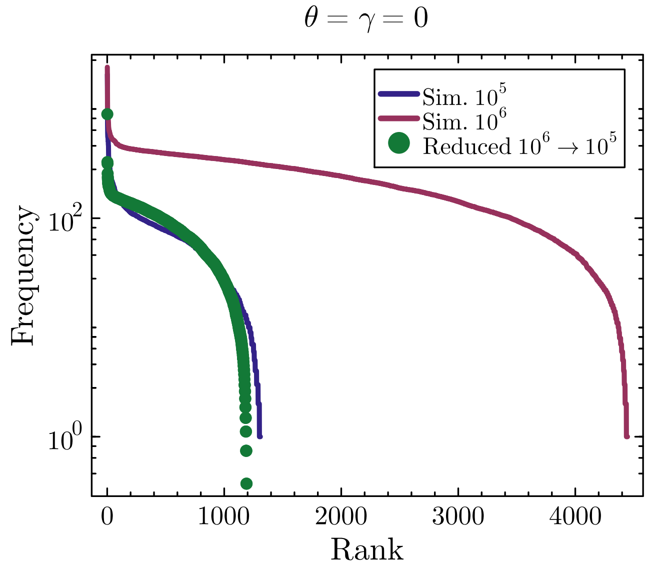

Fig. S2. Example of the reduction method for simulations of the model with $\gamma = \theta = 0$ and $p_0 = 10$. The method is designed to capture changes in the diversity (i.e., the length of the tail) and the frequencies of functions once the distribution is collapsed to a smaller size. The two solid lines show the simulation result for system sizes 10^5 (blue) and 10^6 (red) respectively. The scatter plot shows the rank-frequency distribution that results from the method in this section, namely reducing the rank-frequency distribution at size 10^6 such that it captures the structure at size 10^5.

5. Additional Modeling and Calibration Results

Below we show the fits between the calibrated model and the data for four examples of each system, in Figure S3 for the prokaryotic cells, in Figure S4 for the federal agencies, and in Figure S5 for the cities. In each case, parameter values from the calibration procedure are included in the figure legends, along with a description of the system that is plotted. In Figure S6 we show the behavior of $p(\theta, N)$ for changes in various parameter values in the system, to give an intuitive understanding of the relationship between $p(\theta, N)$, θ, γ and N.

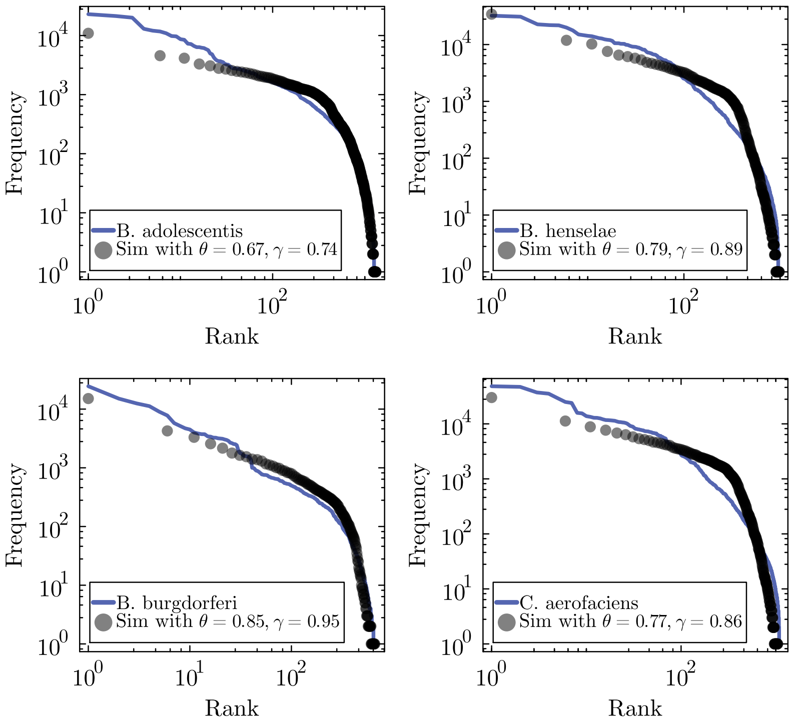

Fig. S3. Sample of four prokaryotic rank-frequency distributions predicted by the model and compared against the data.

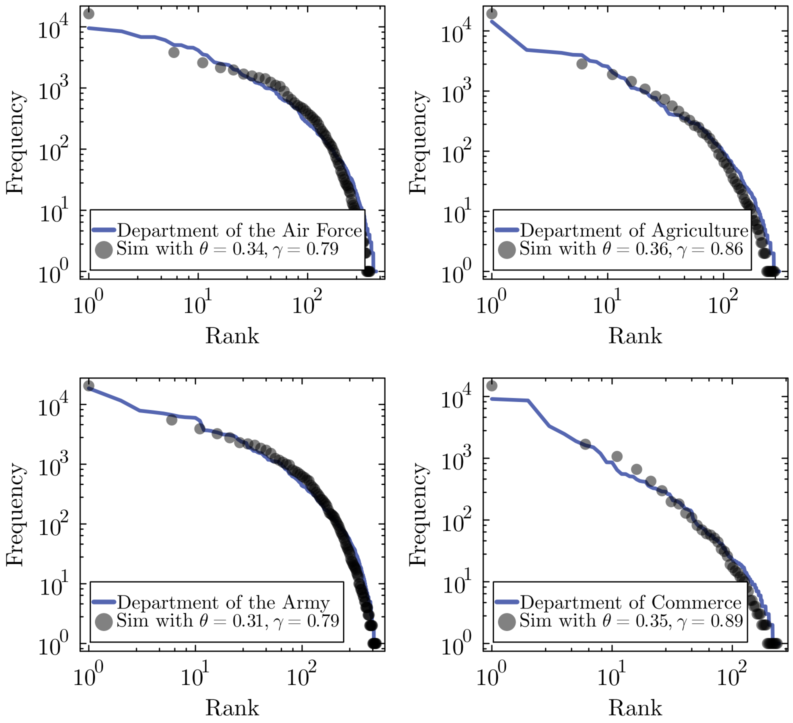

Fig. S4. Sample of four federal agency rank-frequency distributions predicted by the model and compared against the data.

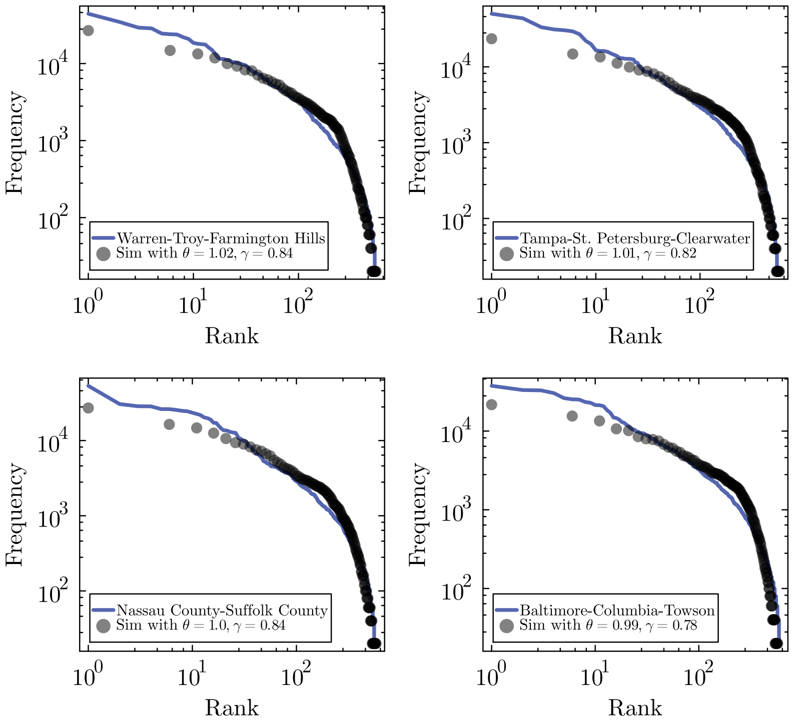

Fig. S5. Sample of four city rank-frequency distributions predicted by the model and compared against the data.

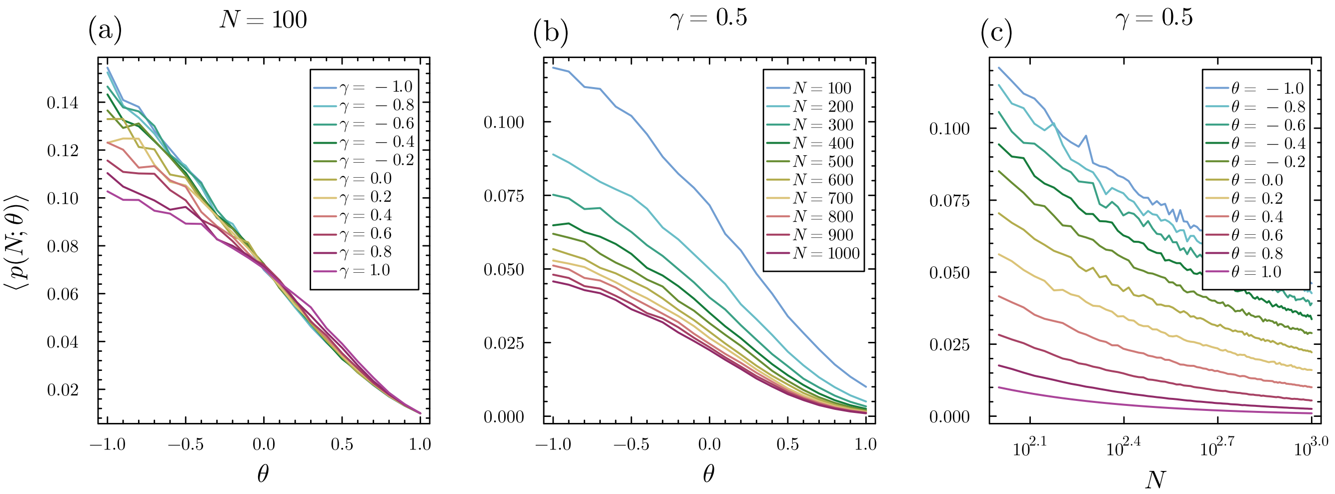

Fig. S6. The probability of new function addition (averaged over 100 realizations, represented by $\langle\cdot\rangle$) as a function of θ, γ and N (for $p_0 = 1$). (a) For a fixed value of $N = 100$ we have plotted the average value of p, $\langle p(N,\theta)\rangle$, against θ for a range of γ values. (b) For a fixed value of $\gamma = 0.5$ we have plotted $\langle p(N,\theta)\rangle$ across θ for a range of N values. (c) For a fixed value of $\gamma = 0.5$ we have plotted $\langle p(N,\theta)\rangle$ against N for a range of θ values. In all cases we see a monotonic decrease of $p(N,\theta)$ against increasing θ, γ and N.

6. Robustness Analyses

A. Diversity Scaling of Urban Areas Across Multiple Years. We conducted a robustness test of the logarithmic scaling observed in cities by analyzing data from multiple years. Specifically, we used data from 2016 and 2015—two years adjacent to our primary analysis year of 2017—as well as 2010, which is the furthest back year that still shares the same SOC code definition as the 2017 data. The results are presented in Fig. S7, with the best-fit coefficients (for $D = b \log N + c$) and R-square values reported in Table S2. Although there are minor variations in the best-fit parameters across years, the logarithmic scaling behavior of cities remains robust over time.

Table S2. Regression coefficients and R^2 values by year for robustness test on additional years of urban system data

Year	b	c	r-sq
2016	116	-1033	0.977
2015	117	-1043	0.976
2010	115	-1013	0.977

B. Stability of Estimated Parameters over Multiple Years of Data. We collected annual data for U.S. metropolitan areas from 2010 to 2017 and evaluated how the model's estimated parameters change over time.

Although occupational data for U.S. metropolitan areas are publicly available dating back to 1997, the classification system used—Standard Occupational Classification (SOC)—has undergone major revisions in 2000, 2010, and 2018 (see: https://www.bls.gov/soc/ for details). These changes complicate comparisons across different SOC regimes. To avoid confounding effects from classification updates and economic disruptions such as COVID-19, we focus on the uninterrupted 2010–2017 period.

First, we select the four largest metropolitan areas and repeat the calibration procedure described in the main text for each year within this period. If the estimated parameters θ and γ remain stable across years within a city, then this provides evidence that θ and γ do not change throughout the evolution of a city, and the dynamic model can be a good representation of the cross-sectional data. The calibrated values over time are shown in Fig. 5 of the main text. This is indeed what we find—θ and γ vary little, and the variation of parameters across cities is generally greater than the variation of parameters across time for the same city. This validates the use of our dynamical model to describe cross-sectional data.

C. Verify Model's Dynamic Predictions Using Longitudinal Data. We perform a robustness analysis to verify the ability of our model to capture the longitudinal dynamics directly. We focus on the largest metro area, New York. We use the data of 2010 as the initial condition of our model, and simulate our model with the value of θ and γ from the original calibration in the main text. We compare the model's prediction of the rank-frequency distribution at different sizes N through the evolution trajectory directly with the data of New York at the same sizes. Note that the size of New York changed noticeably throughout this data set (working population is 4.8 million in 2010, and 6.6 million in 2017). The comparison between the model's predicted distribution and the actual distribution across years is shown in Fig. S8, and the model closely replicates the evolution of the rank-frequency distribution for this period.

D. Robustness Analysis of the Calibration Procedure. We assess the robustness of the calibration results with respect to (i) data noise, (ii) initial conditions and (iii) alternative assumptions (e.g., the type of optimizer used).

We assess the robustness of our calibration procedure using the data of federal agencies, the system class that expresses the greatest diversity in the variation of θ. This variation allows us to easily assess whether variations in (i)-(iii) above lead to significant deviations in θ, and hence the diversity scaling of each federal agency. As in the main text, we performed the calibration procedure on the largest 20 federal agencies and evolved them from an initial condition given as a federal agency of size $\sim 10^3$. In conducting the robustness analysis we performed the following steps:

(i) To assess the calibration results' robustness against data noise, we introduce data noise by constructing resampled data—each agency's rank-frequency distribution was used as a sampling distribution to produce a slightly perturbed organization. This adds noise to the data.

(ii) To assess the calibration results' robustness against variation in the initial conditions, we chose four distinct federal agencies of size around 1000 as initial conditions. These initial conditions show considerable variation (see Fig. S9). Note that each separate initial condition gives its own prescribed value of p_0 following the procedure outlined in Section D.

(iii) To assess the role of the optimizer, we performed the calibration analysis using adaptive differential evolution (ADE, as previously used), and, in addition, random parameter search. Random parameter search is the most naive optimization algorithm, unlike ADE which is a state-of-the-art optimizer.

In sum, these three perturbations to the calibration procedure allow the algorithm's robustness to be assessed across three critical variations of the procedure. Fig. S10 details the robustness analysis for the case of an adaptive differential evolution optimizer, and Fig. S11 details the robustness analysis for the case of a random search black box optimizer. Because the results of the two figures are very similar, we only discuss Fig. S10 below, for more details on Fig. S11 please see its caption.

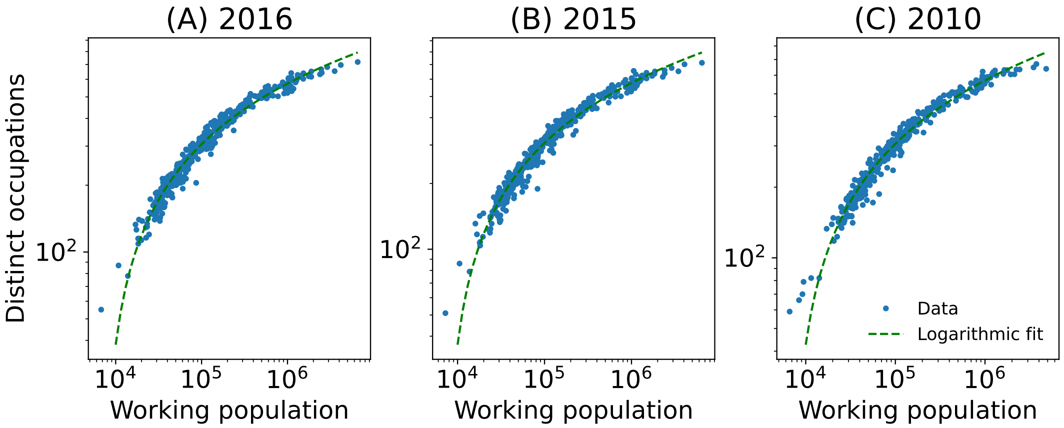

Fig. S7. The scaling relationship between size and function diversity for urban areas in three additional years of data, showing robustness of logarithmic scaling.

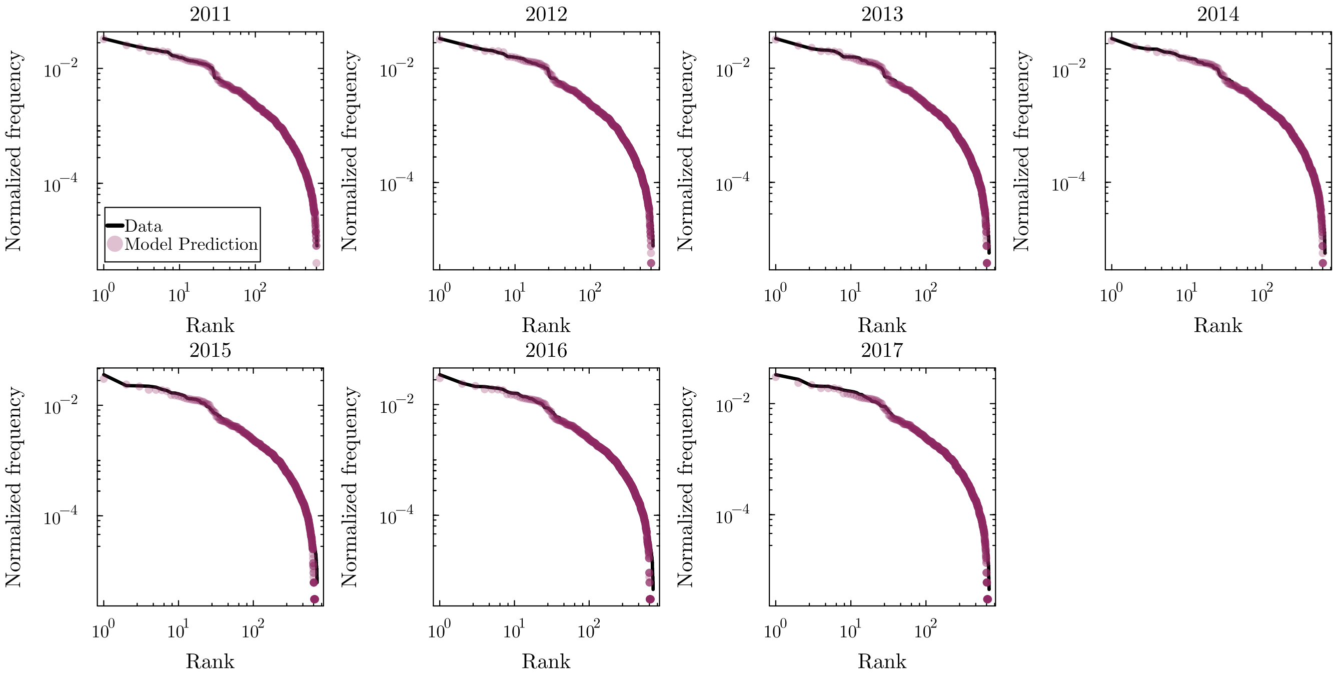

Fig. S8. Comparison of model's dynamic predictions with longitudinal data of New York Metropolitan Area. Taking New York in 2010 as the initial condition, and using the value of θ and γ from the original calibration in the main text, we find that we can closely replicate the evolution of the rank-frequency distributions through time.

In each of the following calibrations, we use a different set of resampled data. Fig. S10(a) shows the estimated θ and γ for four calibrations using four different initial conditions (horizontal axis) for the four largest federal agencies. The estimated parameter values are stable across these perturbations. Panel (b) shows the histogram of all 20 estimated θ values across the four initial conditions. Panel (c) shows the same for estimated γ values. While there are small differences in the extreme values of these distributions, the typical values estimated are similar across these perturbations. Panel (d) shows the γ and θ values in phase space across the four calibrations for the four largest federal agencies. We find that the inter-agency variation in θ and γ is much greater than the variation across different perturbations for the same federal agency, demonstrating the robustness of our calibration procedure.

References

1. M Wang, CJ Herrmann, M Simonovic, D Szklarczyk, C von Mering, Version 4.0 of paxdb: protein abundance data, integrated across model organisms, tissues, and cell-lines. *Proteomics* **15**, 3163–3168 (2015).
2. Q Huang, D Szklarczyk, M Wang, M Simonovic, C von Mering, Paxdb 5.0: Curated protein quantification data suggests adaptive proteome changes in yeasts. *Mol. & Cell. Proteomics* **22** (2023).
3. BJ Shuter, J Thomas, WD Taylor, AM Zimmerman, Phenotypic correlates of genomic DNA content in unicellular eukaryotes and other cells. *The Am. Nat.* **122**, 26–44 (1983).
4. M Simon, F Azam, Protein content and protein synthesis rates of planktonic marine bacteria. *Mar. Ecol. Prog. Ser.* pp. 201–213 (1989).
5. CP Kempes, L Wang, JP Amend, J Doyle, T Hoehler, Evolutionary tradeoffs in cellular composition across diverse bacteria. *The ISME J.* **10**, 2145–2157 (2016).
6. JI Arroyo, C Kempes, An algorithm for predicting per-cell proteomic properties. *bioRxiv* pp. 2024–12 (2024).
7. LM Bettencourt, J Lobo, D Helbing, C Kühnert, GB West, Growth, innovation, scaling, and the pace of life in cities. *Proc. Natl. Acad. Sci.* **104**, 7301–7306 (2007).
8. J Holehouse, et al., A generative model of function growth explains hidden self-similarities across biological and social systems. *arXiv preprint arXiv:2509.14468* (2025).
9. Y Wang, HX Li, T Huang, L Li, Differential evolution based on covariance matrix learning and bimodal distribution parameter setting. *Appl. Soft Comput.* **18**, 232–247 (2014).
10. R Feldt, Blackboxoptim.jl (https://github.com/robertfeldt/BlackBoxOptim.jl) (2018).
11. DT Gillespie, Stochastic simulation of chemical kinetics. *Annu. Rev. Phys. Chem.* **58**, 35–55 (2007).
12. Y Cao, DT Gillespie, LR Petzold, Efficient step size selection for the tau-leaping simulation method. *The J. Chem. Phys.* **124** (2006).

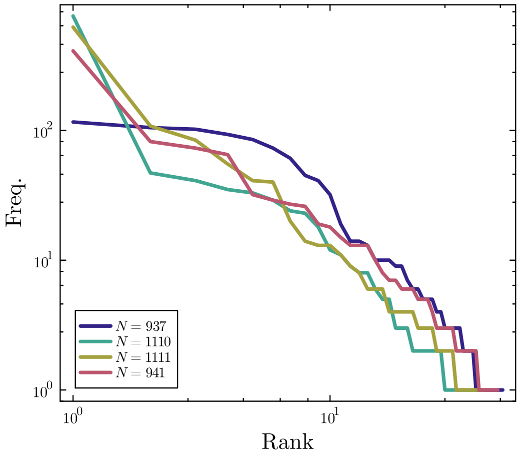

Fig. S9. Rank-frequency distributions of the four initial conditions used in the robustness analysis for the calibrations on the federal agencies. Values of N in the legend state the number of employees in each initial condition.

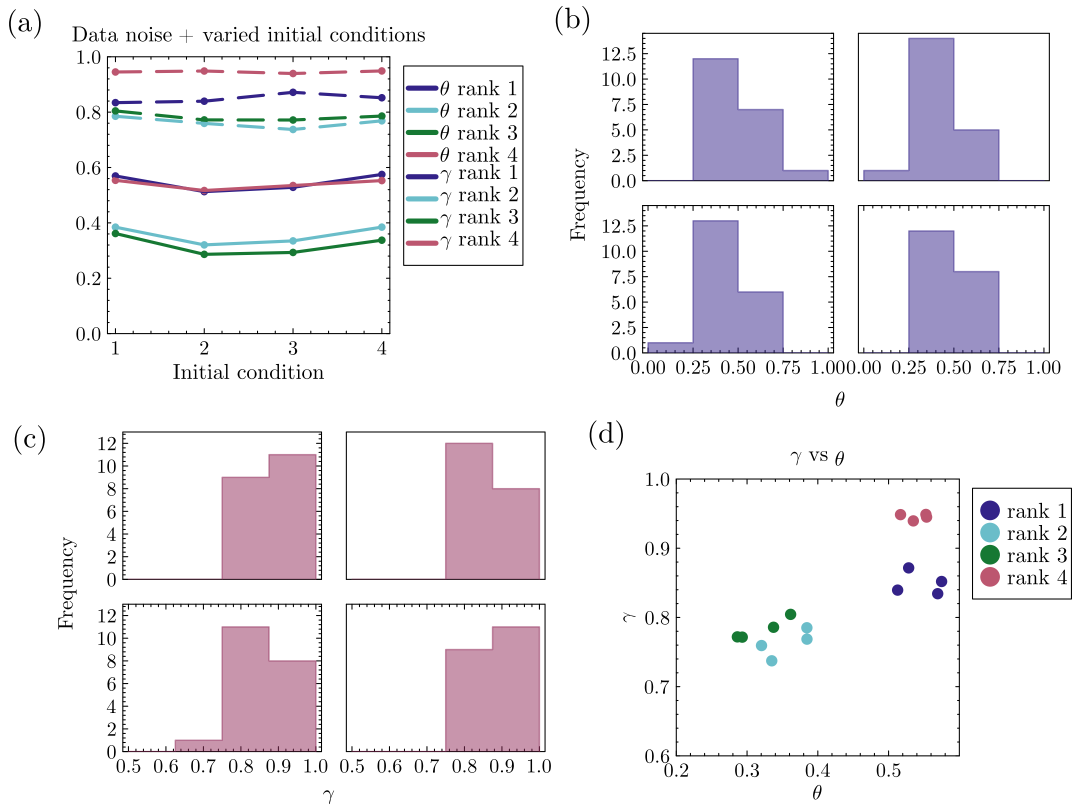

Fig. S10. The results of the calibration analysis using adaptive differential evolution as the optimizer. (a) For the largest four federal agencies we show the variations in θ and γ explicitly over the four different initial conditions. Although small variation is present, each value of θ and γ is generally not highly dependent on the initial condition. (b) Histograms of the 20 values of θ across all federal agencies in the calibration and across the four initial conditions. (c) Histograms of the 20 values of γ across all federal agencies in the calibration and across the four initial conditions. (d) For the largest four federal agencies, we show the variation in the predicted pairs of θ and γ is small, and that inter-agency variation in θ and γ is much greater than the variation in θ and γ across different initial conditions for the same federal agency. In each calibration, a different set of resampled data is used, injecting data noise.

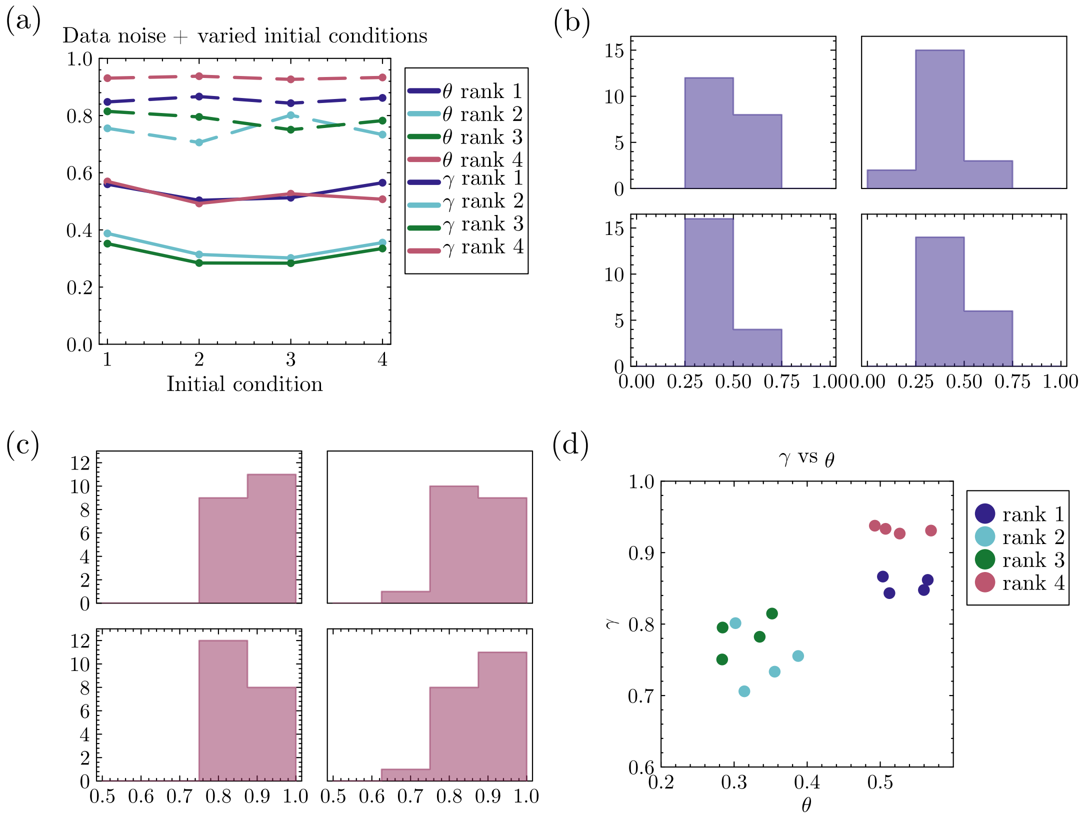

Fig. S11. The results of the calibration analysis using random search as the optimizer. Most of the results remain the same, implying that the choice of optimizer has little impact on the values of θ and γ found.
